# Impact of a Pilot School-Based Nutrition Intervention on Dietary Knowledge, Attitudes, Behavior and Nutritional Status of Syrian Refugee Children in the Bekaa, Lebanon

**DOI:** 10.3390/nu10070913

**Published:** 2018-07-17

**Authors:** Marwa Diab El Harake, Samer Kharroubi, Shadi K. Hamadeh, Lamis Jomaa

**Affiliations:** 1 Department of Nutrition and Food Sciences, Faculty of Agriculture and Food Sciences, American University of Beirut, P.O. Box 11-0.236, Riad El Solh, Beirut 11072020, Lebanon; md106@aub.edu.lb (M.D.E.H.); sk157@aub.edu.lb (S.K.); 2 Environment and Sustainable Development Unit, Faculty of Agriculture and Food Sciences, American University of Beirut, P.O. Box 11-0.236, Riad El Solh, Beirut 11072020, Lebanon; shamadeh@aub.edu.lb; 3 Refugee Health Program, Global Health Institute, American University of Beirut, Beirut 11072020, Lebanon

**Keywords:** nutrition, knowledge, refugees, children, school intervention, Lebanon

## Abstract

This study evaluated the impact of a 6-month school nutrition intervention on changes in dietary knowledge, attitude, behavior (KAB) and nutritional status of Syrian refugee children. A quasi-experimental design was followed; Syrian refuge children in grades 4 to 6 were recruited from three informal primary schools (two intervention and one control) located in the rural Bekaa region of Lebanon. The intervention consisted of two main components: classroom-based education sessions and provision of locally-prepared healthy snacks. Data on household socio-demographic characteristics, KAB, anthropometric measures and dietary intake of children were collected by trained field workers at baseline and post-intervention. Of the 296 school children enrolled, 203 (68.6%) completed post-intervention measures. Significant increases in dietary knowledge (β = 1.22, 95% CI: 0.54, 1.89), attitude (β = 0.69, 95% CI: 0.08, 1.30), and body mass index-for-age-z-scores (β = 0.25, 95% CI = 0.10, 0.41) were observed among intervention vs. control groups, adjusting for covariates (*p* < 0.05). Compared to the control, the intervention group had, on average, significantly larger increases in daily intakes of total energy, dietary fiber, protein, saturated fat, and several key micronutrients, *p* < 0.05. Findings suggest a positive impact of this school-based nutrition intervention on dietary knowledge, attitude, and nutritional status of Syrian refugee children. Further studies are needed to test the feasibility and long-term impact of scaling-up such interventions.

## 1. Introduction

Conflicts and forced displacement are among the main challenges facing our world today, with more people being displaced by conflicts than any other time since World War II. According to the United Nations High Commission for Refugees (UNHCR), a record number of 65.6 million people were uprooted from their homes by conflict and persecution at the end of 2016, 22.5 million of whom were refugees [1]. A refugee is defined as an individual who “owing to a well-founded fear of being persecuted for reasons of race, religion, nationality, membership of a particular social group or political opinion, is outside the country of his nationality, and is unable or unwilling to avail himself of the protection of that country” [2].

Refugees face tremendous challenges that affect their safety, health, livelihoods, and survival with food and nutrition insecurity being considered as one of their basic challenges. Yet, refugee children represent a particularly vulnerable population group that can suffer from the adverse consequences of conflicts, poverty and food insecurity. Studies have shown that the lack of consistent access to safe and nutritious food can have serious detrimental and long-lasting effects on the physical, cognitive, and psycho-social development of children [3,4,5,6]. Children in food insecure households and with poor dietary intakes may suffer from nutrient deficiencies, increased illnesses, poor general health, and increased cognitive and behavioral problems that can affect not only their educational attainment, but also their economic productivity later in life [7].

Despite the high vulnerability of refugee children to the adverse consequences of food insecurity and poverty, few studies to date were conducted to explore the health and nutritional status of refugee children in complex emergencies and protracted crises [8,9,10]. In fact, the limited research conducted within crisis-settings focuses primarily on assessing the prevalence of malnutrition among young children (<5 years), such as stunting, wasting, and anemia [11], with less emphasis being placed on the nutritional status of school-aged children. Recent evidence highlights the importance of considering older children, who may be equally, if not more, vulnerable to the adverse consequences of poverty and food insecurity compared to their younger siblings [7,12,13]. In addition, school children are at increased risk of poor dietary behaviors, including skipping breakfast, consuming low amounts of fruits and vegetables, and adopting unhealthy snacking behaviors that can affect their nutritional status and expose children to higher risk of weight gain and associated co-morbidities [14,15]. Although the first 1000 days of the child’s life remain a critical period to ensure adequate growth and development [16,17,18], recent studies have shown that school-aged children and adolescents have the potential for catch-up growth, which makes them a suitable age group to target through well-designed nutrition interventions [19,20].

Schools can offer an optimal setting to reach out to a large number of children and to promote healthy eating habits and lifestyle behaviors through classroom-based nutrition education, role modeling of heathy behaviors and offering of nutritious snacks and meals [21,22]. Scientific evidence supports the effectiveness of multi-component, school-based interventions in improving the dietary and health-related knowledge and attitude of primary school-aged children [23,24,25]; however, evidence is less consistent in terms of the impact of such interventions on children’s dietary behaviors [26,27]. A previous review on the effectiveness of school feeding programs in developing countries emphasized the importance of integrating interventions that include nutrition and health educational components to complement the provision of nutritious food and snacks within feeding programs to help alleviate hunger and improve the children’s micronutrient status [28]. Despite the strong evidence supporting the positive impact of school-based interventions, the effectiveness of such interventions in improving the nutritional status of vulnerable children within conflict and displacement settings has not been adequately explored [9].

Today, the Syrian refugee crisis represents one of the largest humanitarian disasters worldwide with 5.5 million Syrian individuals fleeing the country since 2011 and seeking refuge in neighboring countries, including Turkey, Jordan, and Lebanon [29]. Lebanon is a small middle income country in the Middle East and North Africa region (MENA) region that has the highest per capita concentration of refugees worldwide [30,31,32]. As of April 2018, slightly less than a million Syrian individuals were registered with the UNHCR in Lebanon as refugees, of which, 36% (354,326 individuals) reside in the Bekaa, a rural and underdeveloped region that is geographically close to the Syrian–Lebanese border [33]. The high influx of Syrian refugees to the impoverished host communities in the Bekaa have added further strain to the limited resources and basic services available, including access to adequate food, water, shelter, education, and health services [34,35].

This study aimed to evaluate the impact of a 6-month pilot school-based nutrition intervention on changes in dietary knowledge, attitude, and behavior of Syrian refugee children enrolled in informal primary schools located in the rural region of the Bekaa in Lebanon. A secondary objective of the study was to explore the effect of the intervention on the dietary intake and nutritional status of children. Findings from this pilot project can provide the evidence for conducting multi-component interventions that aim at alleviating food insecurity and improving the nutritional status and health outcomes of children residing in low-income, conflict-affected settings.

## 2. Materials and Methods

### 2.1. Study Design and Population

This was a quasi-experimental study conducted as part of a larger two year-project called ‘GHATA: Bringing Education to Informal Tented Settlements’. The GHATA project aimed to design three modular schools that provide Syrian refugee children aged 6 to 14 years with a credentialed education and adequate nutrition through offering healthy snacks and nutrition education. These informal schools served as spaces for provision of education and child protection programs targeting Syrian refugees living in some of the most underserved communities in the Bekaa. As of 2016, it has been estimated that more than 250,000 children, approximately half of school-aged Syrian children registered in Lebanon, are out of school [36], and only 15% of refugee children in the Bekaa valley are enrolled in schools [37].

The informal modular schools built within the GHATA project welcomed refugee children who dropped out of school due to the war in Syria and assisted in overcoming some of the main reported barriers limiting Syrian refugee children from enrolling within the Lebanese public school system. These barriers included the cost of education, the need for child labor, limited capacities for registration in schools, language and social integration difficulties [36]. Schools were structurally designed by the Center for Civic Engagement at the American University of Beirut (AUB) and were managed by the Kayany Foundation, a local non-governmental organization that provides education to disadvantaged Syrian refugee children.

For the purpose of this study, two of the three informal schools were randomly selected to serve as intervention schools and the third served as a control school. Schools had similar school enrollment capacities and community characteristics (such as the number of public schools in the area and number of hospitals). Each school had on average a total of 640 students enrolled in grades one to six. Each grade level had three sections with an average classroom size of 27 students. The three schools were located approximately 10–14 km away from each other and were situated in the three areas of Majdal Anjar, Saadneyil, and Bar Elias within the Bekaa valley, Lebanon. The intervention conducted in this study focused on school children enrolled in grades 4 to 6 within the three informal schools.

### 2.2. Sample Size and Recruitment

A power calculation was performed prior to the start of the study indicating that at least 64 students (32 students from intervention and 32 students from control) were required to detect a significant difference in dietary knowledge scores (effect size = 2.8) between intervention and control groups with 80% power and 95% confidence interval. The expected effect size was based on results from another school-based nutrition intervention conducted among children enrolled in grades 4 and 5 within public schools in Lebanon [14]. An additional 25% was added to the sample size to account for potential dropouts and incomplete data. The intended sample size was 80 school children, and the final sample enrolled at baseline consisted of 296 children (195 from intervention schools and 101 from control school), please see supportive material Figure S1 (flow diagram for study participants).

The present study was conducted over two academic years (2015–2016 and 2016–2017). Recruitment of the study population took place in September at the beginning of each of the consecutive school years during the registration period. After receiving approval from schools’ administration, the research team approached parents of children enrolled in grades 4 to 6 during the school registration period explaining the purpose of the study. Parents who agreed to participate in the study were contacted to schedule interviews for data collection with children and their mothers at a date of their preference within a private classroom in their respective schools. Schools were considered a convenient site to conduct the interviews as they were located only a walking distance (5–10 min) from the informal tented settlements (refugee camps) where school children and their families reside. All study participants gave their informed consent for inclusion before participating in the study. The study was conducted in accordance with the Declaration of Helsinki, and the protocol was approved by the Ethics Committee of American University of Beirut (NUT.LJ.07).

### 2.3. Description of Intervention

The school-based nutrition intervention implemented in the present study was composed of two main components: (1) delivering health and nutrition education modules on a bi-weekly basis; and (2) providing children with locally-prepared nutritious snacks. Children in grades 4 to 6 within the intervention schools received the combination of both components, whereas children in the control school received their usual curriculum and a standard snack. Children in the control group received all the intervention material at the end of the second year of the project. The research team conducted field trip observations, meetings with school supervisors, and short evaluation surveys with children to assess fidelity to the project. Results from this process evaluation showed good adherence to the intervention components and any challenges were addressed throughout the study duration.

#### 2.3.1. Classroom-Based Educational Sessions

The classroom-based health and nutrition education modules developed in this study were tailored for children in the upper elementary school levels and were based on the social cognitive theory, focusing primarily on observational learning, behavioral capability and self-efficacy. Prior school-based interventions showed modest effectiveness of this theory and its constructs to help increase the cognitive and behavioral skills of children and improve their dietary behaviors both at home and at school [21,38,39]. A total of 12 interactive classroom-based health and nutrition sessions were delivered by classroom teachers on a bi-weekly basis over a period of 6 months. Each of the educational sessions lasted approximately 45 min. Topics covered within these educational sessions included basic hygienic practices, importance of consuming breakfast daily, role of fruits and vegetables in a healthy diet, benefits of consuming water versus sugar-sweetened beverages, healthy snacking behaviors, and importance of physical activity. Hands-on activities and games were incorporated as part of these educational sessions to reinforce the main messages within each module. In addition, school teachers within the intervention schools were given a resource box that included visually-appealing and culturally-sensitive posters and printed material, such as the Lebanese food guide pyramid, the World Health Organization (WHO) poster for an adequate hand washing technique, to be mounted on the walls within the classrooms and throughout the school facilities. These visual aids were intended to reinforce and promote healthy eating behaviors, handwashing, among other basic hygiene practices among children. Intervention schools were also provided with physical activity resources that can be used as part of the physical education and activity sessions, such as skipping ropes, hula hoops, and balls.

To assist teachers with implementing the health and nutrition sessions, two-day training-of-trainers workshops were conducted at the beginning of the school year and a refresher training was conducted mid-year to ensure fidelity to the intervention. During the training workshops, intervention toolkits that included educational lesson plans, games, activities, and other supportive material were provided to all trained school teachers. Teachers were also provided with knowledge, skills, and relevant resources required for effective delivery of the intervention. The toolkit and all educational lesson plans were developed by a team of nutrition experts at the Department of Nutrition and Food Sciences at AUB using a variety of resources including the United States Department of Agriculture (USDA) online resources, age-appropriate science textbooks adopted by the Lebanese public schools, and published material and evidence available from similar low-to-middle-income country (LMIC) settings [12,14,40]. All educational materials were pilot tested with the school teachers at the beginning of the intervention to ensure cultural-sensitivity and appropriateness.

#### 2.3.2. Locally-Prepared Nutritious Snacks

One of the main components of the GHATA project was to establish small kitchens or cooking units within the three newly established schools and to provide training to kitchen employees hired from the local Syrian refugee community to prepare nutritious and safe snacks to children during the school year. Children in the intervention group were provided with one snack item on a daily basis during the school break according to a pre-planned weekly menu. Food availability and children acceptability were taken into consideration by the research team when planning for the weekly snack menus. Snacks consisted of cheese or ‘labneh’ sandwiches, spinach pies, or thyme pastries (known as ‘manakeesh’). In addition, children were offered fruits (oranges, apples, or bananas) twice a week, depending on seasonality, availability, and cost. The snack offered to children in the intervention schools supplied on average 357 kcal per day, 11 g protein, 58 g carbohydrates, and 9 g of fat. Dietary needs of children for calcium, iron, vitamins A and C were also considered as part of the snack planning and composition using the Dietary Reference Intakes (DRI) as a reference. Children in the control group also received standard daily snacks that consisted of thyme pastries, a locally-acceptable and affordable choice offered in public schools in the country. The standard snack supplied children on average with 294 kcal per day, 4.9 g protein, 31 g carbohydrates, and 17 g of fat. Children in the intervention and control schools found the offered snacks to be favorable and consumed them regularly. Only few children in the intervention schools expressed a preference to spread cheeses rather than locally-produced white cheese varieties at the beginning of the study, and were thus offered alternative sandwich options. It is worth noting that shortly afterwards, children expressed content with the offered white cheese options after noticing their peers in the school consuming and enjoying these varieties. All children in the intervention and control groups received daily snacks, regardless of their involvement in the study.

### 2.4. Data Collection (Instruments and Outcomes)

Face-to-face interviews with children and their mothers, who served as proxy respondents, were conducted by a team of trained field workers at the beginning of each of the academic years (September–October; 2015–2016 and 2016–2017) to collect baseline data. Measurements included sociodemographic characteristics of households, dietary knowledge, attitude and behavior (KAB) of children, as well as children’s anthropometric measurements and dietary intake. Measurements of KAB, anthropometrics, and dietary intake were repeated at the end of each of the school years (May–June). Interviews lasted on average 45 min.

#### 2.4.1. Socio-Demographic Characteristics

Information on child’s gender and age, maternal age, educational level, employment, income, access to food assistance, and living conditions of household were obtained during interviews with the mother of the child. The crowding index (CI) is a proxy measure of household socio-economic status that has been previously used in Lebanon and other LMIC settings providing reliable results [41,42,43]. CI was calculated as the total number of household members divided by the total number of rooms in a household (excluding kitchens, bathrooms and balconies) [41]. Household food security status was also assessed using an Arabic-translated, locally validated version of the Household Food Insecurity Access Scale (HFIAS) [43]. The HFIAS consisted of nine questions that ask about modifications households made in their diet or food consumption patterns due to limited resources to acquire food, each of which can be answered as ‘No’, ‘Rarely’, ‘Sometimes’, and ‘Often’ with an individual score of “0”, “1”, “2”, and “3”, respectively. This 9-question scale produces a total score between 0 and 27 with higher scores indicating higher food insecurity [44].

#### 2.4.2. Knowledge, Attitude and Behavior (KAB)

The dietary knowledge, attitude and behavior (KAB) of children were assessed at baseline and at 6 months follow-up using a 32-item questionnaire. The questionnaire included three sections: (1) nutrition knowledge section (15 questions); related to the importance of breakfast, fruits, vegetables and water consumption, healthy snacking, benefits of being physically active; (2) nutrition attitude section (10 questions); it included statements about healthy eating and making healthy food choices; and (3) dietary and lifestyle behaviors’ section (7 questions), such as frequency of fruits, vegetables, dairy consumption, snacking, skipping meals and watching TV. The KAB-related questions were either formulated or adapted from published questionnaires and school-based studies conducted in Lebanon [14] and other middle-income country settings [12,40,45]. All questions were reviewed by one academic coordinator and 3 school teachers from the participating schools and were also pre-tested with 20 school children (aged 11–14 years) from the local Syrian refugee community to ensure accuracy, clarity and cultural-adequacy. Minor modifications were made to the questions according to feedback from children and teachers.

The questions on dietary knowledge, attitude and behaviors were separately analyzed. For the nutrition knowledge questions, each correct response was allocated a score of 1 point and an incorrect or no response was allocated 0 point. The total knowledge score ranged between 0 and 15 points with higher scores indicating the child displayed better nutritional knowledge. For the attitude statements, each question was measured on a 3-point Likert scale (1 = I agree, 2 = I am not sure, 3 = disagree) using faces that expressed these scales to make it easier for children to report their answers. The 10 attitude statements were also summed into a single score (range 0–10); whereby the favorable options (I agree) were given 1 point each and unfavorable options (I am not sure or disagree) were given 0 point. A higher attitude score reflected more positive attitude towards healthy eating. For questions related to dietary and lifestyle behaviors, favorable options were also given a score of 1 and unfavorable options were given a score of 0. The total behavior score ranged between 0 and 22 points with a higher score reflecting good dietary and lifestyle behaviors of children. Details related to the number of options for each of the KAB questions and the coding of correct answers are presented as supporting material (Table S1).

#### 2.4.3. Dietary Intake

The dietary intake of children was assessed at baseline and at follow up by trained nutritionists using the 24-h dietary recall approach. Mothers were present at the time of the interview and served as proxy respondents. Interviewers followed the 5-steps USDA multiple pass method when collecting data on the food, beverage, and snack intake of children during the previous 24-h period or any other typical day during that week. Only few children reported dietary intake from a day different to that of the previous day. An atypical day included limited food availability at home for the child or the child was fasting for religious purposes during a month different than the fasting month of ‘Ramadan’, typically observed by Muslims. The steps included (1) the quick list; (2) the forgotten foods list; (3) time and occasion at which foods were consumed; (4) the detail cycle; and (5) the final probe [46]. To assist children and their mothers in assessing the portion sizes and amounts of food consumed at home and at school by the child, two-dimensional portion size posters, household measures and graduated food models were used (Millen and Morgan, Nutrition Consulting Enterprises, Framingham, MA, USA). Daily energy, macronutrient and micronutrient intake of children were computed from the 24-h recalls using the food composition database of the Nutritionist Pro software (Nutritionist Pro, Axxya Systems, San Bruno, CA, USA, version 5.1.0, 2018). The software food database was expanded by adding analyses of locally consumed foods and recipes [47]. Given that there are no gender- or age-specific DRIs for Middle Eastern populations, values arising from the analyzed data were compared to the US-based DRIs for children, as recommended by the Institute of Medicine [48].

#### 2.4.4. Anthropometric Measures

Anthropometric measurements of children (weight, height, waist circumference) and their mothers (weight and height) were obtained by the trained nutritionists. Measurements were carried out only at baseline for mothers and at baseline and follow up for children using standard protocols and equipment. Weight was measured to the nearest 0.1 kg in light indoor clothing and with bare feet or stockings, using a portable standard calibrated balance (Seca model 877, Germany) and height was measured, without shoes, to the nearest 0.1 cm using a portable stadiometer (Seca, model 213, Hamburg, Germany). A non-stretchable measuring tape (Seca model 201, Germany) was used to measure waist circumference of children at the level of the umbilicus to the nearest 0.1 cm, with the subject standing and after normal expiration. All measurements were taken twice and the average of the 2 values was reported. Body mass index (BMI) (kg/m^2^) was calculated by dividing the weight (kg) over the height squared (m^2^). Mothers were categorized as normal, overweight or obese based on the WHO classifications [49]. Age and gender-specific BMI z-scores (BAZ), height for age z-scores (HAZ) and weight for age z-scores (WAZ) were calculated for children using the WHO Anthro Plus software (1.0.4) [50]. Children were classified as thin, normal weight, overweight, and obese based on the WHO age and gender-specific cut-offs [51]. Stunting and underweight were defined as HAZ < –2 SD and WAZ < –2 SD of the WHO child growth standards median, respectively [51]. In addition, the Waist to height ratio (WHtR) was calculated by dividing waist circumference (WC) by height, both measured in centimetres [52]. The cut-off point of ≥0.5 was used to identify children with elevated WHtR, an indicator of abdominal obesity among children [52,53].

### 2.5. Data Analysis

Data were entered and analyzed using Stata/SE version 12 (StataCorp., College Station, TX, USA). Descriptive statistics were performed and presented as means and standard error (SE) for continuous variables or as frequencies and proportions for categorical variables. At baseline, comparisons of sociodemographic and anthropometric characteristics of participants between intervention and control groups were assessed using clustered independent *t*-tests and chi-square analyses (stata command clttest and clchi). Paired *t*-tests were used to compare independently the differences in KAB scores, energy and nutrient intakes between baseline and 6-months follow up within each of the intervention and control groups (within-group differences). In addition, between-group differences (intervention versus (vs.) control groups) in mean changes of scores (i.e., follow-up minus baseline scores) were evaluated using clustered independent *t*-tests. Additionally, two-way analysis of variance was conducted to test differences in mean changes of KAB scores and anthropometric measurements between intervention/control and school year. No significant interactions were found between group status and school year with mean change in dietary knowledge, attitude or behavior scores and with anthropometric measurements (*p* > 0.05). Multiple linear regression analyses were conducted to test the effect of the nutrition intervention on mean changes in dietary KAB and anthropometric measures (BMI for age *z*-score (BAZ), waist to height ratio (WHtR), HAZ, and WAZ) among children, adjusting for covariates found significantly different at baseline between intervention and control groups. In case two variables were highly correlated (*p*-value < 0.001), then one of the variables was excluded from the model to avoid multicollinearity. Linear regressions were performed using the wild cluster bootstrap-t procedure (stata command cgmwildboot) [54,55]. This procedure provides adequate power and desirable false rejection rates for performing statistical inference, even for data with small numbers of clusters [55]. Sensitivity analyses were conducted to determine whether our conclusions were biased by incomplete data. We accounted for missing values, which were assumed to be missing at random, using a simulation-based statistical technique (namely, multiple imputations). Imputed models showed that the majority of results were similar to the complete case analyses; see Tables S2 and S3 in supportive material. A *p*-value of <0.05 was considered statistically significant.

## 3. Results

Survey data were collected at baseline from 296 school children out of 318 that were contacted to take part in the study (93% response rate). Data at baseline and at follow up was available for 203 children (completion rate = 68.6%). The sample size was reduced to *n* = 183 due to clustering effect. At baseline, the mean age of children was 11.04 ± 0.23 years with an approximately equal gender distribution among the study sample (50.7% females and 49.3% males). Almost a third of their mothers had intermediate level education or more, whereas 42% of fathers had similar educational levels. The majority of mothers (94.4%) and 43% of fathers were unemployed. Approximately 63% of households reported an average monthly income less than 200 USD and the majority received some form of assistance in the past three months, either in the form of food baskets, e-card, or as conditional cash. Significant socio-demographic and anthropometric differences were observed between the intervention and control groups at baseline (*p* < 0.05) and are presented in Table 1. No significant differences were noted between intervention and control groups at baseline with respect to dietary knowledge (range 0–15) and behavior scores (range 0–22). However, dietary attitude scores (range 0–10) were found to be on average significantly higher at baseline among children in the intervention compared to the control group (8.18 ± 0.18 vs. 7.47 ± 0.23, *p* = 0.047).

Table 2 presents between-group differences (intervention versus control) with respect to mean change from baseline to 6-months follow up in dietary knowledge, attitude and behavior scores as well as anthropometric measures of children in the study sample. Compared to the control group, a greater change in knowledge scores was observed among the intervention group (2.25 ± 0.22 vs. 0.89 ± 0.24, *p* = 0.002). Similarly, greater changes in BAZ and HAZ scores were noted among children in the intervention compared to control groups, (0.10 ± 0.06 vs. −0.10 ± 0.08, *p* = 0.039 and 0.39 ± 0.04 vs. 0.24 ± 0.05, *p* = 0.024). No other significant differences in mean changes of attitude, behavior, WAZ scores and WHtR were noted between intervention and control groups. Within-group differences in KAB and anthropometric measures were presented in Table S4, as supplementary material.

Using linear regression models, the mean change in dietary knowledge scores increased on average by 1.22 units (95% CI = 0.54, 1.89; *p* < 0.001) among children in the intervention compared to the control group, even after adjusting for other covariates (Table 3). Similarly, the mean change in dietary attitude scores increased by 0.69 units (95% CI = 0.08, 1.30, *p* = 0.026) in the intervention group within the regression model, while the mean change in behavior scores was not found to be significantly different by group status (intervention vs. control) (see Table 3).

As for the anthropometric measures, there was a significant increase in mean BAZ scores among children in the intervention compared to the control group (β = 0.25, 95% CI =0.10, 0.41; *p* = 0.001). No other significant differences were noted between intervention and control groups in the regression models with respect to WAZ, HAZ, or WHtR (see Table 4).

Between-group differences (intervention vs. control) with respect to mean change of energy intake and macro-and micro-nutrients are presented in Table 5. Compared to the control group, children in the intervention group had on average significantly higher mean changes in daily intakes of total energy (kcal), dietary fiber, protein, and saturated fat (*p* < 0.05). In addition, children in the intervention group had significantly higher mean changes in intakes of key micronutrients, including vitamin K (*p* < 0.001), zinc (*p* = 0.037), calcium (*p* = 0.017), and magnesium (*p* = 0.007). It was also noted that between-group differences for vitamin E and phosphorus approached statistical significance (*p* = 0.05). Within-group differences were also noted in the study sample and were presented in supportive material (Table S5).

## 4. Discussion

The present study evaluated the impact of a pilot school-based nutrition intervention on the dietary knowledge, attitude, behaviors and nutritional status of refugee children enrolled in primary-level informal schools. To the best of our knowledge, this is the first school-based nutrition intervention that combines nutrition education with provision of healthy snacks among primary-level school children residing in refugee camp settings.

Findings from this pilot nutrition intervention suggest significant improvements in the dietary knowledge and attitude of children post-intervention, even after adjusting for other covariates. These results are consistent with findings from previous school-based interventions conducted among primary-school children in LMIC settings [12,14,38,59]. The positive results in the present study could be explained by the adoption of a theory-driven approach in our intervention, mainly the social cognitive theory, when developing the nutrition and health educational curriculum. Examples are the use of evidence-base constructs suggested to be effective in changing dietary knowledge and behaviors [60,61], such as observational learning through the role modeling of healthful behaviors by school teachers and promoting behavioral capability and self-efficacy among children through games and activities that build the confidence of children to identify what are healthy meal and snack choices, and how to not skip breakfast and engage in fun and culturally-acceptable physical activities within their context. In addition, the use of a multi-component approach that included classroom-based health and nutrition education combined with provision of healthy snacks could have contributed to the positive increases in children’s knowledge scores and to improvement in their attitude scores.

On the other hand, mean change in behavior scores was not found to be significantly different between children from the intervention and control groups in the regression models. This finding was not surprising as other school-based studies conducted in LMIC settings have shown mixed results with respect to behavioral changes and outcomes. The HEALTH-E-PALS, a multi-component school-based pilot intervention conducted in Lebanon among primary-level school children (9–11 years), showed significant improvements in the nutritional knowledge, self-efficacy, and children’s dietary behaviors, such as daily breakfast intake and lower consumption of chips and sweetened drinks, yet limited to no significant increases in fruits and vegetables intake or in the children’s physical activity-related behaviors. Similarly, Francis et al., 2010 reported reductions in intakes of sugar, fat and salt-dense snacks and sodas, but no significant impact on the physical activity level of primary-school children enrolled in a short-term school-based nutrition and physical activity intervention in Trinidad and Tobago [38]. On the other hand, the HealthKick, a primary school nutrition intervention conducted in low-income schools in Western Cape Province (South Africa), showed no significant improvements in the snacking behaviors or the overall diet quality of grade 4 children [27].

Differences in results between our study and others may be attributed to a number of factors, such as variations in intervention components, duration, or mode of delivery of intervention (by school teachers vs. dedicated research team members or expert dietitians). Evidence in this field also shows that nutrition education may improve the knowledge of children towards healthy eating and active lifestyles; however, changing behaviors is a much more complex process that may be influenced by a multitude of economic, environmental, cultural and social factors [39,62,63]. In the case of the present study, it is worth considering the unique study population and the harsh context where the school intervention was conducted within informal tented settlements (refugee camps). In fact, baseline results show that the majority of children’s households in the study sample were experiencing severe forms of food insecurity, had low socioeconomic status, and were dependent on some form of humanitarian assistance. The poor living conditions of Syrian refugee children in parallel with the limited resources available for their families within a refugee camp setting [64,65] can make it reasonably challenging for children to access adequate food resources or make healthy dietary and lifestyle choices, despite the significant improvements in their dietary knowledge. Other factors that may hinder the ability of children to make significant behavioral changes may be related to their psychological and mental health status. Most school-based interventions conducted within similar refugee settings report the high levels of anxiety, fear, and depression experienced by children after fleeing a war [26,27,66], in addition to the daily environmental stressors that they are exposed to in the host country making healthier dietary choices more challenging and less attainable [67,68].

Our pilot intervention showed improvements in the dietary intake of refugee children as assessed using the 24-h recall data. Compared to the control group, children in the intervention group had on average significantly larger increases in intakes of total energy (kcal), dietary fiber, protein, and saturated fats, as well as larger increases in intakes of vitamin K, zinc calcium, and magnesium. These dietary changes may be attributed to the healthy snacks provided to children during the school days. In fact, the snacks contributed on average to a 357 kcal per day, representing 16% of daily dietary needs of children in this age group, while also meeting their daily needs for several micronutrients, including calcium, iron, vitamins A and C (11–77% of dietary reference intakes). Although the mean change in intake of saturated fats was significantly higher among children in the intervention compared to the control group, this increase may be explained by the consumption of full-fat dairy products, which are rich in saturated fats. Nevertheless, dairy products represent a rich source of protein and micronutrients including calcium, phosphorus and B-vitamins, and these products were not frequently consumed among refugee children in the study sample. In addition, when examining the intake of saturated fats as percent of total daily energy intake at follow up among children in the intervention group, it was found to contribute to 7.2% of energy intake, which is still below the WHO upper limits for saturated fat in this age group [69].

Findings from the present study showed a significant increase in mean BAZ scores among children in the intervention group compared to control, even after adjusting for other covariates. Nevertheless, the mean changes in WHtR (an indicator of abdominal obesity) [52,53] was not found to be statistically significant between the intervention and control groups. In fact, when considering within-group analyses, results showed that the proportion of overweight children did not differ significantly between baseline and follow up within the intervention (20.5% vs. 21.4%) or control groups (12.5% vs. 9.1%), rather the proportion of thin children decreased slightly in the intervention group (1.8% to 0.8%) while it increased in the control group (3.4% to 4.5%), see supportive material Table S6. Although, HAZ and WAZ scores were not found to be statistically significant between the intervention and control groups in the complete case analysis, a larger increase in these scores could have been detected with a larger sample size, as observed in the imputed models (Tables S2 and S3). These results suggest that the intervention did not contribute to an increase in overweight and obesity levels among children in the study sample, but rather to a potential improvement in their overall growth. This increase could be explained by increases in energy intake among children receiving the healthy snacks as part of the school intervention or it can be attributed to improvements in some of the dietary behaviors of children post-intervention, even though the overall behavior scores did not reach statistical significance. A previously published Cochrane review that examined the effectiveness of obesity-prevention interventions supported the beneficial impact that programs conducted in schools can have on BMI of children, particularly targeting those aged 6–12 years [70]. Interventions that were found to be promising in that review were those that included a school curriculum addressing healthy eating and physical activity, improving the nutritional quality of foods offered at schools and teacher involvement, many of which were covered in the present study.

The present study has a number of strengths. This is the first study, to the best of our knowledge, evaluating the impact of a multi-component school-based nutrition intervention on dietary knowledge, behaviors and nutritional status of primary-level school children residing in refugee camp settings. The sustainability of the intervention beyond the study duration was considered through training school teachers on the educational curriculum and the establishment of cooking units within schools and training kitchen employees to plan and prepare safe and nutritious snacks for children during the school year. Another strength of the study is the overall acceptability and satisfaction of children with the health and nutrition educational sessions and daily snack choices as assessed through field observations, student evaluations, and meetings with teachers and school administration.

Although the findings of this pilot intervention were promising, the study has a number of limitations that need to be considered. First, the study was conducted in one area of the country with a small number of schools that may not be representative of the entire refugee population. Differences between intervention and control groups were also noted at baseline and may have biased the results. However, adjustments for all covariates found to be statistically significant between both groups were done in the regression models. Lessons learned from this pilot intervention can provide insights for future studies to be conducted in similar vulnerable population groups in order to reduce potential selection bias between intervention and control groups. For example, the duration of the refugees’ stay in the host country and their place of residence (rural or urban context) prior to migration are important to consider when setting the inclusion and exclusion criteria, as these factors may contribute to their socioeconomic, cultural background and potential differences. Another limitation of the study is the use of a single 24-h recall to estimate dietary intake, which may have been subject to recall bias. Nevertheless, every attempt was exerted to minimize recall bias through collecting dietary data from children in the presence of their mothers (as proxy respondents). In addition, interviewers were trained nutritionists who received extensive training to reduce judgmental verbal and non-verbal communication and underwent a 2-day training workshop to ensure the standardization of data collection protocol. The research team also faced several challenges when conducting this intervention, such as the high turnover of teachers in schools due to their refugee status and their relocation into other areas in Lebanon or immigration to other countries. Nevertheless, training of new teachers was conducted throughout the school year, in addition to the pre-scheduled training and refresher workshops at the beginning and middle of the academic year to ensure fidelity to the intervention protocol. Another limitation was the high attrition rate (31.4%) noted in the study and that can be attributed to the low attendance of children at the end of the school year when post-intervention visits were scheduled, as children were skipping school days to assist their families in agriculture-related activities during the harvest season. Nevertheless, sensitivity analyses showed that the majority of results were still similar between the complete case and imputed model analyses.

In conclusion, findings from the present study suggest a promising impact of a combined nutrition education and healthy snack intervention on the dietary knowledge and nutritional status of refugee primary-school children. Future studies are needed to test the feasibility of scaling up such nutrition interventions within low-income and conflict-affected settings and to evaluate their long-term impact on children’s overall health and nutritional status.

## Figures and Tables

**Table 1:** Baseline socio-demographic and anthropometric characteristics of school-aged children enrolled in intervention and control elementary schools in the Bekaa region, Lebanon (*n* = 203).

	Total Sample (*n* = 203)	Intervention (*n* = 114)	Control (*n* = 89)	*p*-Value ^1^
**Socio-demographic characteristics**				
Child’s age (years), Mean ± Standard Error (SE)	11.04 ± 0.23	11.16 ± 0.31	10.89 ± 0.47	0.651
Child’s gender, *n* (%)				
Males	100 (49.3)	58 (50.9)	42 (47.2)	0.458
Females	103 (50.7)	56 (49.1)	47 (52.8)	
Mother’s age (years), Mean ± SE	35.75 ± 0.41	36.16 ± 0.67	35.25 ± 0.75	0.395
Mother’s education, *n* (%)				0.0001
No school	51 (25.9)	18 (16.2)	33 (38.4)	
Primary school	76 (38.6)	37 (33.3)	39 (45.3)	
≥Intermediate school	70 (35.5)	56 (50.5)	14 (16.3)	
Mother’s employment, *n* (%)				0.910
Unemployed	186 (94.4)	105 (94.6)	81 (94.2)	
Employed	11 (5.6)	6 (5.4)	5 (5.8)	
Father’s education, *n* (%)				0.003
No school	35 (17.9)	11 (10.0)	24 (27.9)	
Primary school	78 (39.8)	45 (40.9)	33 (38.4)	
≥Intermediate school	83 (42.3)	54 (49.1)	29 (33.7)	
Father’s employment, *n* (%)				0.715
Unemployed	84 (43.1)	47 (42.7)	37 (43.5)	
Employed	111(56.9)	63 (57.3)	48 (56.5)	
Monthly income (USD dollars), *n* (%)				0.754
<200	121 (62.7)	70 (64.8)	51 (60.0)	
200–399	61 (31.6)	31 (28.7)	30 (35.3)	
≥400	11 (5.7)	7 (6.5)	4 (4.7)	
Crowding index ^2^, Mean ± SE	5.83 ± 0.53	4.72 ± 0.26	7.14 ±0.28	0.0004
Household Food insecurity score, Mean ± SE	15.37 ± 0.71	14.31 ± 0.83	16.64 ± 1.09	0.132
Household Food Insecurity status ^3^, *n* (%)				0.021
Non-severely food insecure	41 (20.9)	30 (27.3)	11 (12.8)	
Severely food insecure	155 (79.1)	80 (72.7)	75 (87.2)	
Receive assistance (Yes), *n* (%)	161 (82.6)	88 (80.0)	73 (85.9)	0.250
Food assistance: food basket ^4^ (Yes), *n* (%)	43 (22.1)	17 (15.5)	26 (30.6)	0.010
Food assistance: e-card ^5^ (Yes), *n* (%)	152 (77.9)	80 (72.7)	72 (84.7)	0.069
Conditional cash ^6^ (Yes), *n* (%)	12 (6.2)	6 (5.5)	6 (7.1)	0.670
**Anthropometric characteristics ^7^**				
**Mothers (*n* = 171)**				
Body Mass Index (BMI) (kg/m^2^), Mean ± SE	29.66 ± 0.35	29.09 ± 0.59	30.33 ± 0.64	0.197
BMI status ^8^, *n* (%)				0.189
Normal (≤24.9 kg/m^2^)	35 (18.5)	22 (21.4)	13 (15.1)	
Overweight (25.0–29.9 kg/m^2^)	67 (35.4)	39 (37.9)	28 (32.6)	
Obese (≥30 kg/m^2^)	87 (46.0)	42 (40.8)	45 (52.3)	
**Children**				
Weight (kg), Mean ± SE	35.62 ± 1.37	37.24 ± 1.62	33.58 ± 2.34	0.239
Height (cm), Mean ± SE	141.63 ± 1.70	143.86 ± 1.98	138.82 ± 2.89	0.193
Waist Circumference (cm), Mean ± SE	67.27 ± 1.03	68.22 ± 1.43	66.10 ± 2.02	0.412
BMI for Age *Z*-score (BAZ), Mean ± SE	0.03 ± 0.06	0.11 ± 0.10	−0.06 ± 0.12	0.297
BAZ, *n* (%)				0.524
Thin (BAZ ≤ −2)	5 (2.5)	2 (1.8)	3 (3.4)	
Normal (−2 < BAZ ≤ +1)	162 (80.6)	87 (77.7)	75 (84.3)	
Overweight (+1 < BAZ ≤ +2)	22 (10.9)	14 (12.5)	8 (9.0)	
Obese (BAZ > +2)	12 (6.0)	9 (8.0)	3 (3.4)	
Waist to Height ratio (WHtR), Mean ± SE	0.48 ± 0.003	0.48 ± 0.006	0.48 ± 0.007	0.926
WHtR, *n* (%)				
WHtR < 0.5	142 (75.1)	76 (71.0)	66 (80.5)	
WHtR ≥ 0.5 (elevated)	47 (24.9)	31 (29.0)	16 (19.5)	
Height for age *Z*-score (HAZ), Mean ± SE	−0.40 ± 0.11	−0.16 ± 0.11	−0.71 ± 0.12	0.011
HAZ, *n* (%)				0.438
HAZ ≥ −2	178 (88.6)	100 (89.3)	78 (87.6)	
HAZ < −2 (stunted)	23 (11.4)	12 (10.7)	11 (12.4)	
Weight for age *Z*-score (WAZ) ^9^, Mean ± SE	−0.003 ± 0.21	0.31 ± 0.39	−0.34 ± 0.58	0.418
WAZ, *n* (%)				0.538
WAZ ≥ −2	74 (96.1)	37 (97.4)	37(94.9)	
WAZ < −2 (underweight)	3 (3.9)	1 (2.6)	2 (5.1)	
**Knowledge Attitude Behavior (KAB) scores ^10^**				
Knowledge scores, Mean ± SE	9.85 ± 0.14	10.06 ± 0.20	9.69 ± 0.22	0.260
Attitude scores, Mean ± SE	7.88 ± 0.20	8.18 ± 0.18	7.47 ± 0.23	0.047
Behavior scores, Mean ± SE	9.07 ± 0.46	9.82 ± 0.53	8.31 ± 0.71	0.133

^1^ Comparison of baseline characteristics between intervention and control groups was conducted for continuous and for categorical variables using clustered independent and chi-squared tests. Statistical significance was determined at *p*-value <0.05. ^2^ Crowding index: the average number of people per room, excluding the kitchen and bathroom. ^3^ Households were grouped into four levels of food insecurity: food secure (2.6%); mildly food insecure (2.5%); moderately food insecure (15.8%); and severely food insecure (79.1%). ^4^ World Food Programme (WFP) distributes a food basket tailored to beneficiaries’ nutrition needs, preferences, activity levels and other factors (climate condition, demographic profile, and existing levels of malnutrition and disease [56]. ^5^ WFP provides electronic vouchers that allow beneficiaries to purchase food at local shops [57]. ^6^ Cash transfers given to stimulate beneficiaries to invest in their health, nutrition and education [58]. ^7^ Anthropometric measurements of mothers and children were categorized based on World Health Organization (WHO) classification [49,51]. ^8^ Mothers with underweight BMI (*n* = 3) were added to the normal BMI group. ^9^ Weight for age z-scores were assessed only for children ≤10 years old (*n* = 68) as per the WHO growth charts [51]. ^10^ The total knowledge, behavior and attitude scores ranged: 0–15 points, 0–10 points and 0–22 points, respectively.

**Table 2:** Between-group differences (intervention versus control) in mean change of nutrition knowledge, attitude, behavior scores and anthropometric measures of school-aged children (*n* = 183).

	Intervention (*n* = 102)	Control (*n* = 81)	*p*-Value ^1^
	Mean Change ± SE		
**Knowledge Attitude Behavior (KAB) scores ^2^**			
Knowledge scores	2.25 ± 0.22	0.89 ± 0.24	0.002
Attitude scores	0.97 ± 0.17	0.82 ± 0.19	0.294
Behavior scores	0.36 ± 0.72	0.43 ± 0.96	0.521
**Anthropometric measurements**			
BMI for Age *Z*-score (BAZ)	0.10 ± 0.06	−0.10 ± 0.08	0.039
Waist to Height ratio (WHtR)	−0.01 ± 0.008	−0.02 ± 0.008	0.260
Height for age *Z*-score (HAZ)	0.39 ± 0.04	0.24 ± 0.05	0.024
Weight for age *Z*-score (WAZ) ^3^	0.32 ± 0.12	0.08 ± 0.18	0.177

^1^ Differences in mean changes (follow up minus baseline) of KAB scores and anthropometric measures between intervention and control groups were conducted using clustered independent tests. Statistical significance was determined at *p*-value < 0.05. ^2^ The total knowledge, behavior and attitude scores ranged: 0–15 points, 0–10 points and 0–22 points, respectively. ^3^ Weight for age z-scores were assessed only for children ≤10 years old (*n* = 68) as per the WHO growth charts [51].

**Table 3:** General linear regression models for mean change in nutrition knowledge, attitude and behavior scores among school-aged children in the study sample ^†^ (*n* = 183).

	Mean Change in Knowledge Scores Adjusted β (95% CI)	Mean Change in Attitude Scores Adjusted β (95% CI)	Mean Change in Behavior Scores Adjusted β (95% CI)
Group status (Intervention)	1.22 (0.54, 1.89), *p* < 0.001	0.69 (0.08, 1.30), *p* = 0.026	−0.20 (−2.53, 2.14)
School year (Year 2)	0.26 (−0.63, 1.15)	−0.63 (−1.07, −0.19), *p* = 0.005	0.37 (−0.99, 1.73)
Child’s age	−0.18 (−0.49, 0.13)	0.02 (−0.24, 0.28)	1.56 (0.45, 2.68), *p* = 0.006
Gender (Females)	0.04 (−0.60, 0.68)	0.33 (−0.26, 0.93)	0.80 (−1.06, 2.65)
Mother’s education			
No school (Ref.)	---	---	---
Primary	−0.19 (−1.04, 0.65)	0.02 (−0.80, 0.84)	−1.54 (−3.82, 0.75)
Intermediate to higher	0.02 (−0.89, 0.93)	−0.17 (−0.97, 0.62)	−1.73 (−4.28, 0.83)
Father’s education			
No school (Ref.)	---	---	---
Primary	−0.54 (−1.14, 0.06)	−0.29 (−0.98, 0.40)	−1.23 (−3.50, 1.03)
Intermediate to higher	−1.59(−2.14, −1.05), *p* < 0.001	−0.54 (−1.32, 0.23)	−0.07 (−2.39, 2.25)
Crowding Index	−0.01 (−0.17, 0.15)	0.12 (0.01, 0.24), *p* = 0.040	0.05 (−0.25, 0.35)
Food basket (Yes)	−0.84 (−1.38, −0.30), *p* = 0.002	0.67 (−0.01, 1.36)	1.50 (−0.68, 3.69)
Household food insecurity status			
Non-severely food insecure (Ref.)	---	---	---
Severely food insecure	−0.40 (−1.13, 0.32)	0.19 (−0.35, 0.74)	2.20 (0.93, 3.48), *p* = 0.001
Height for age *Z*-score (HAZ)	0.23 (−0.01, 0.46)	−0.12 (−0.36, 0.13)	0.53 (−0.02, 1.08)

^†^ Variables adjusted for in the three models testing the impact of group status were variables found significantly different at baseline between intervention group (IG) and control group (CG). These variables include school year, child’s age, mother‘s and father’s educational levels, crowding index, receiving assistance (food basket), household food insecurity status, and children’s anthropometric measures (HAZ).

**Table 4:** General linear regression models for mean change in anthropometric measurements (BAZ, WHtR, HAZ, and WAZ) among school-aged children in the study sample ^1^ (*n* = 183).

	Mean Change in BAZ Adjusted β (95% CI)	Mean Change in WHtR Adjusted β (95% CI)	Mean Change in HAZ Adjusted β (95% CI)	Mean Change in WAZ ^2^ Adjusted β (95% CI)
Group status (Intervention)	0.25 (0.10, 0.41), *p* = 0.001	0.02 (−0.01, 0.05)	0.19 (−0.07, 0.45)	0.25 (−0.04, 0.54)
School year (Year 2)	−0.05 (−0.14, 0.04)	0.01 (−0.03, 0.05)	−0.11 (−0.24, 0.02)	−0.05 (−0.11, −0.003), *p* = 0.039
Mother’s education				
No school (Ref.)	---	---	---	---
Primary	0.01 (−20, 0.22)	0.02 (−0.06, 0.11)	0.10 (−0.80, 0.28)	0.02 (−0.13, 0.17)
Intermediate to higher	−0.04 (−0.22, 0.13)	−0.002 (−0.07, 0.06)	0.06 (−0.30, 0.42)	0.04 (0.02, 0.06), *p* < 0.001
Father’s education				
No school (Ref.)	---	---	---	---
Primary	−0.17 (−0.35, −0.001), *p* = 0.048	−0.02 (−0.07, 0.03)	−0.04 (−0.34, 0.26)	−0.15 (−0.22, −0.07), *p* < 0.001
Intermediate to higher	−0.17 (−0.39, 0.06), *p* < 0.001	−0.002 (−0.06, 0.05)	0.01 (−43, 0.44)	−0.16 (−0.23, −0.10), *p* < 0.001
Crowding Index	−0.01 (−0.03, 0.01)	0.002 (−0.05, 0.01)	0.01 (−0.02, 0.05)	−0.002 (−0.02, 0.01)
Food basket (Yes)	0.05 (−0.21, 0.30)	0.02 (−0.02, 0.06)	0.07 (−0.02, 0.16)	−0.004 (−0.17, 0.16)
Household food insecurity status				
Non-severely food insecure (Ref.)	---	---	---	---
Severely food insecure	0.02 (−0.10, 0.15)	0.008 (−0.01, 0.03)	0.06 (−0.14, 0.26)	0.01 (−0.07, 0.09)

^1^ Variables adjusted for in the three models testing the impact of group status were variables found significantly different at baseline between IG and CG. These variables include school year, mother‘s and father’s educational levels, crowding index, receiving assistance (food basket), and household food insecurity status. ^2^ Weight for age z-scores were assessed only for children ≤10 years old (*n* = 68) as per the WHO growth charts [51].

**Table 5:** Between-group differences (intervention versus control) in mean change of total energy (kcal/day) and macro-and micronutrient intakes (g/day) among school-aged children (*n* = 183).

Intervention (*n* = 102) Control (*n* = 81)			
	**Mean Change** **Mean ± SE**		***p*-Value ^†^**
Energy intake (Kcal)	94.71 ± 68.80	−110.00 ± 77.02	0.0469
**Macronutrients**			
Carbohydrates (g)	9.76 ± 9.89	−8.31 ± 11.07	0.132
Sugar (g)	−1.36 ± 3.63	0.99 ± 4.07	0.661
Dietary fiber (g)	1.03 ± 1.01	−2.60 ± 1.19	0.027
Protein (g)	5.95 ± 1.94	−0.38 ± 2.17	0.033
Total fat (g)	3.25 ± 4.60	−8.35 ± 5.17	0.064
MUFA (g)	1.76 ± 3.49	−5.45 ± 4.76	0.130
PUFA (g)	−6.45 ± 1.07	−2.04 ± 1.25	0.212
Saturated fat (g)	1.48 ± 0.82	−1.76 ± 0.92	0.017
Trans fat (g)	−0.05 ± 0.04	0.03 ± 0.05	0.893
**Micronutrients**			
Vitamin C (mg)	10.41 ± 5.27	0.08 ± 5.90	0.116
Vitamin A (µg)	90.31 ± 53.18	−0.05 ± 69.22	0.167
Vitamin D (µg)	0.34 ± 0.20	0.12 ± 0.22	0.242
Vitamin E (mg)	0.12 ± 0.65	−1.70 ± 0.75	0.055
Vitamin K (µg)	124.08 ± 20.49	−36.90 ± 24.87	0.0001
Vitamin B1 (mg)	0.10 ± 0.08	−0.0001 ± 0.08	0.196
Vitamin B2 (mg)	0.08 ± 0.18	−0.01 ± 0.22	0.375
Vitamin B3 (mg)	1.36 ± 0.79	−0.03 ± 0.88	0.140
Vitamin B6 (mg)	0.11 ± 0.05	−0.01 ± 0.06	0.094
Vitamin B12 (µg)	0.06 ± 0.24	0.24 ± 0.30	0.676
Folate (µg)	12.41 ± 24.10	−19.97 ± 32.24	0.224
Iron (mg)	0.94 ± 1.21	−2.40 ± 1.65	0.073
Zinc (mg)	0.30 ± 0.55	−1.58 ± 0.70	0.037
Calcium (mg)	141.19 ± 40.61	−28.75 ± 50.59	0.017
Phosphorus (mg)	73.11 ± 34.11	−23.73 ± 38.20	0.050
Magnesium (mg)	26.76 ± 9.32	−18.29 ± 10.43	0.007
Sodium (g)	0.25 ± 0.13	0.03 ± 0.14	0.143
Potassium (g)	0.18 ± 0.10	−0.03 ± 0.12	0.109

^†^ Differences in mean changes of nutrient intakes between intervention and control groups were conducted using clustered independent tests. Statistical significance was determined at *p*-value < 0.05.

## Supplementary Materials

The following are available online at http://www.mdpi.com/2072-6643/10/7/913/s1, Figure S1: Flow diagram for study Participants; Table S1: Dietary and lifestyle-related knowledge attitude and behavior (KAB) questionnaire and coding criteria; Tables S2 and S3: Imputed general linear regression models for mean change in nutrition knowledge, attitude and behavior scores among school-aged children in the study sample (*n* = 296); Table S4: Within-group differences (baseline versus follow up) in mean change of nutrition knowledge, attitude, behavior scores and anthropometric measures of school-aged children (*n* = 183); Table S5: Within-group differences (baseline versus follow up) in mean change of total energy (kcal/day) and macro- and micronutrient intakes (g/day) among school-aged children (*n* = 178); Table S6: Post-intervention nutritional status of school-aged children enrolled in intervention and control elementary schools in the Bekaa region, Lebanon (*n* = 183).
