# Supplementary material for: Impact of a Pilot School-Based Nutrition Intervention on Dietary Knowledge, Attitudes, Behavior and Nutritional Status of Syrian Refugee Children in the Bekaa, Lebanon
Source: Nutrients. 2018 Jul 17;10(7):913. doi: 10.3390/nu10070913 (PMC6073287; doi:10.3390/nu10070913)
Supplement: Supplementary file 1 [file nutrients-10-00913-s001.zip › supplementary material/FigureS1.docx]

Three informal primary-schools in the Bekaa, Lebanon

Children eligible for study over 2 school years (n=1458)

Children contacted to take part in study (n=318)

Consent and assent forms signed by mothers and their children (n=296)

**Intervention**

**(2 schools)**

**Control**

**(1 school)**

Data collected from children at baseline (n=101)

- Knowledge, attitude, behavior
- 24 hour dietary recall
- Anthropometrics
- Socio-economic characteristics(mother as proxy respondent)
- )

Data collected from children at baseline (n=195)

- Knowledge, attitude, behavior
- 24 hour dietary recall
- Anthropometrics
- Socio-economic characteristics(mother as proxy respondent)

Number of children lost to follow up (n=12)

- Dropped out of school (n=8)
- Did not show up (n=4)

Number of children lost to follow up (n=81)

- Dropped out of school (n=26)
- Did not show up (n=26)
- Migration/immigration (n =29)

Data collected from children 6- months post-intervention (n=89)

- Knowledge, attitude, behavior
- 24 hour dietary recall
- Anthropometrics

Data collected from children 6- months post-intervention (n=114)

- Knowledge, attitude, behavior
- 24 hour dietary recall
- Anthropometrics

**Figure S1** Flow diagram for study participants
