# Supplementary material for: Impact of a Pilot School-Based Nutrition Intervention on Dietary Knowledge, Attitudes, Behavior and Nutritional Status of Syrian Refugee Children in the Bekaa, Lebanon
Source: Nutrients. 2018 Jul 17;10(7):913. doi: 10.3390/nu10070913 (PMC6073287; doi:10.3390/nu10070913)
Supplement: Supplementary file 1 [file nutrients-10-00913-s001.zip › supplementary material/Table S1.docx]

**Table S1.** Dietary and lifestyle Knowledge attitude and behavior (KAB) evaluation items

| **Knowledge (0-15)** |
| --- |
| 1. Fruits and Vegetables are part of a healthy diet^§^. 2. Yes 3. No |
| 1. It is important to eat different kinds of fruits every week^§^. 2. Yes 3. No |
| 1. Eating breakfast is an important part of a healthy lifestyle^§^. 2. Yes 3. No |
| 1. Is snacking important between meals^§^? 2. Yes 3. No |
| 1. An example of a healthy snack is _________^ƾ^? 2. Fruits 3. Chocolate 4. Candies 5. Chips 6. Soft drinks |
| 1. Which of the following are healthy choices? circle the correct answers¤  \| 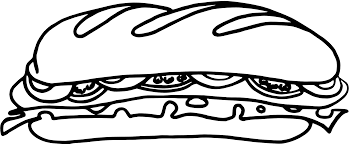a. \| 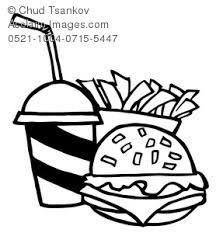d. \| 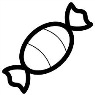g. \| \| --- \| --- \| --- \| \| 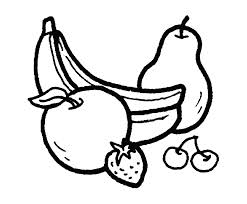b. \| 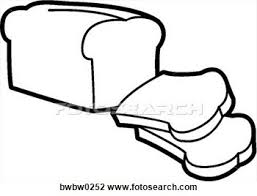e. \| 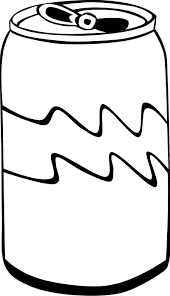h. \| \| 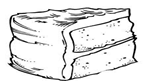c. \| 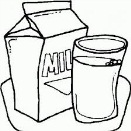f. \| 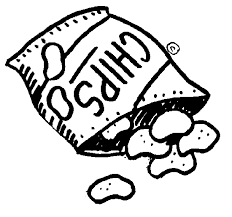i. \| |
| 1. From which type of food should you eat least¶? 2. Bread, rice and pasta 3. Milk, cheese and yogurt 4. Sweets, fats and oils 5. Fruits and vegetables 6. Meat , chicken and eggs 7. I don’t know |
| 1. How many servings of fruits and vegetables should you have per day^¥^? 2. One 3. 2-3 4. 4-5 5. 5 or more 6. I don’t know |
| 1. Eating breakfast helps me do well at school^£^. 2. Yes always 3. Sometimes 4. Never |
| 1. The best fluid for my body is : (choose one answer)€ 2. Water 3. Sweetened juice 4. Soft drinks 5. All of the above 6. I don’t know |
| 1. Sweetened bottled/canned juice and soft drinks cause dental caries^§^. 2. Yes 3. No 4. I don’t know |
| 1. Chocolate and candies cause dental caries^§^. 2. Yes 3. No 4. I don’t know |
| 1. Eating a lot of sweets makes me gain weight^§^. 2. Yes 3. No 4. I don’t know |
| 1. Which activity is better for your health₾?   a. Sitting and watching TV b. Engaging in any type of sports  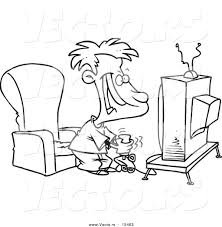  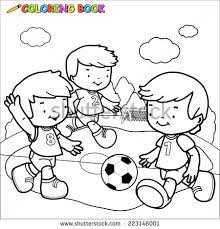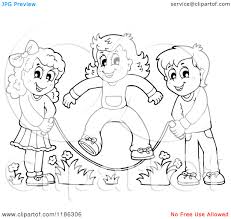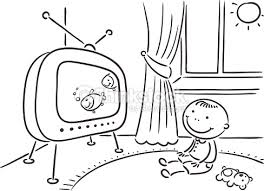 |
| 1. Being physically active is important because……₼ 2. It keeps you healthy 3. It gives you energy 4. It makes you happy 5. Improves your academic performance 6. All of the above |
| § Yes coded as “1”, No and I don’t know coded as “0”.  ƾ Fruits coded as “1”, chocolate, candies, chips and soft drinks coded as “0”.  ¤ a,b,e,f coded as “1”, any other combination of responses coded as “0”.  ¶ Sweets, fats, oils coded as “1”, any other response coded as “0”.  ¥ 2-3 servings of fruits and vegetables per day, any other response coded as “0”.  £ Yes always coded as “1”, Sometimes and Never coded as “0”.  € Water coded as “1”, any other response coded as “0”.  ₾ Engaging in any type of sports coded as “1”, sitting and watching TV coded as “0”.  ₼ All of the above coded as “1”, any other response coded as “0”. |

| **Attitudes (0-10)** |
| --- |
| 1. I think healthy food tastes good |
| 1. I think eating healthy is very important |
| 1. I believe my health in future may be affected by what I eat today |
| 1. I think eating breakfast every day is good for my health |
| 1. Drinking a glass of milk everyday is good for my health |
| 1. I think drinking a glass of water every day is good for my health |
| 1. I think Fruits and vegetables are good for my health |
| 1. I think water is the best fluid for my body |
| 1. I think chips are very high in salt |
| 1. I think soft drinks are very high in sugar |
| For all attitude items, Agree coded as “1”, Not sure or Disagree coded as “0”. |

| **Behaviors(0-22)** |
| --- |
| 1. How often do you eat vegetables¶?    1. Several times a day    2. Once a day    3. 2-3 times/week    4. Once a week    5. Never |
| 2. How often do you eat fruits¶?   - 1. Several times a day   2. Once a day   3. 2-3 times/week   4. Once a week   5. Never |
| 3. How often did you skip meals ^£^?   - 1. Several times a day   2. Once a day   3. 2-3 times/week   4. Once a week   5. Never |
| 4. How often did you drink milk or eat milk products such as Labneh, cheese or yogurt¶?   - 1. Several times a day   2. Once a day   3. 2-3 times/week   4. Once a week   5. Never |
| 5. Over the past month, how often do you watch TV at home ^ƾ?^   1. Everyday 2. 4-6 days/week 3. 2-3 days/week 4. 1 day/week 5. Never |
| 6. Are you able to snack between meals^§^?   - 1. Yes   2. No |
| 7. Are you able to consume 3 meals per day (breakfast, lunch and dinner) ^§^?   - 1. Yes   2. No |
| ¶ Several times a day coded as “4”, Once a day coded as “3”, 2-3 times/week coded as “2”, once a week coded as “1” and Never coded as “0”.  £ Several times a day coded as “0”, Once a day coded as “1”, 2-3 times/week coded as “2”, once a week coded as “3” and Never coded as “4”.  ƾ Everyday coded as “0”, 4-6 days /week coded as “1”, 2-3 days /week coded as “2”, once a week coded as “3” and Never coded as “4”.  § Yes coded as “1”, No coded as “0”. |
