# Supplementary material for: Impact of a Pilot School-Based Nutrition Intervention on Dietary Knowledge, Attitudes, Behavior and Nutritional Status of Syrian Refugee Children in the Bekaa, Lebanon
Source: Nutrients. 2018 Jul 17;10(7):913. doi: 10.3390/nu10070913 (PMC6073287; doi:10.3390/nu10070913)
Supplement: Supplementary file 1 [file nutrients-10-00913-s001.zip › supplementary material/Table S2 and S3.docx]

**Table S2.** Imputed general linear regression models for mean change in nutrition knowledge, attitude and behavior scores among school-aged children in the study sample^†^ (n=296).

|  | Mean change knowledge scores  Adjusted β (95%CI) | Mean change attitude scores  Adjusted β (95%CI) | Mean change behavior scores  Adjusted β (95%CI) |
| --- | --- | --- | --- |
| Group status (Intervention) | 1.51(1.09,1.92), p<0.001 | 0.67(0.28,1.07),p=0.001 | -0.05(-1.38,1.28) |
| School year (Year 2) | 0.36(-0.17,0.89) | -0.47(-0.79,-0.16),p=0.003 | 0.49(-0.42,1.41) |
| Child’s age | -0.21(-0.43,0.01) | -0.03(-0.16,0.11) | 0.94(0.51,1.37),p<0.001 |
| Gender (Females) | -0.01(-0.45,0.44) | 0.09(-0.32,0.51) | -0.10(-1.15,0.95) |
| Mother’s education |  |  |  |
| No school (Ref.) | --- | --- | --- |
| Primary | 0.17(-0.44,0.78) | 0.12(-0.50,0.74) | -0.65(-2.43,1.12) |
| Intermediate to higher | 0.34(-0.12,0.80) | 0.16(-0.41,0.73) | -1.10(-2.36,0.15) |
| Father’s education |  |  |  |
| No school (Ref.) | --- | --- | --- |
| Primary | -0.72(-1.27,-0.16),p=0.012 | -0.23(-0.86,0.41) | -1.21(-2.60,0.17) |
| Intermediate to higher | -1.05(-1.61,-0.48),p<0.001 | -0.58(-1.18,0.01) | 0.004(-1.31,1.32) |
| Crowding Index | 0.01(-0.05,0.07) | 0.10(0.06,0.15),p<0.001 | -0.06(-0.25,0.13) |
| Food basket (Yes) | -0.44(-0.91,0.03) | 0.89(0.67,1.11),p<0.001 | 1.37(-0.15,2.89) |
| Household food insecurity status |  |  |  |
| Non-severly food insecure (Ref.) | --- | --- | --- |
| Severly food insecure | -0.27(-0.99,0.45) | 0.20(-0.08,0.47) | 2.40(1.82,2.98),p<0.001 |
| Height for age Z-score (HAZ) | 0.19(0.04,0.34),p=0.014 | -0.12(-0.30,0.07) | 0.20(-0.22,0.62) |
| † Variables adjusted for in the three models testing the impact of group status were variables found significantly different at baseline between IG and CG. These variables include school year, child’s age , mother and father’s educational levels,crowding index, receiving assistance (food fasket), household food insecurity status, and children’s anthropometric measures (HAZ). | | | |

**Table S3.** Imputed general linear regression models for mean change in anthropometric measurements (BAZ, WHtR, HAZ, and WAZ) among school-aged children in the study sample^†^(n=296).

|  | Mean change in BAZ  Adjusted β (95%CI) | Mean change in WHtR  Adjusted β (95%CI) | Mean change in HAZ  Adjusted β (95%CI) | Mean change in WAZ^¶^  Adjusted β (95%CI) |
| --- | --- | --- | --- | --- |
| **Group status** (Intervention) | **0.24(0.14,0.33),p<0.001** | 0.01(-0.17,0.03) | **0.22(0.07,0.37),p=0.004** | **0.28(0.20,0.35),p<0.001** |
| **School year** (Year 2) | 0.01(-0.05,0.07) | 0.01(-004,0.03) | **-0.18(-0.25,-0.10),p<0.001** | **-0.11(-0.18,-0.04),p=0.001** |
| **Mother’s education** |  |  |  |  |
| No school (Ref.) | --- | --- | --- | --- |
| Primary | -0.01(-0.14,0.11) | **0.03(0.01,0.04),p=0.001** | 0.09(-0.01,0.19) | 0.05(-0.02,0.12) |
| Intermediate to higher | -0.09(-0.20,0.01) | -0.003(-0.02,0.01) | 0.07(-0.08,0.22) | -0.02(-0.11,0.07) |
| **Father’s education** |  |  |  |  |
| No school (Ref.) | --- | --- | --- | --- |
| Primary | -0.06(-0.17,0.05) | **-0.03(-0.05,-0.02),p<0.001** | -0.12(-0.29,0.05) | **-0.10(-0.18,-0.03), p=0.005** |
| Intermediate to higher | **-0.12(-0.24,-0.005),p=0.041** | **-0.01(-0.03,-0.001),p=0.036** | -0.08(-0.28,0.12) | **-0.12(-0.21,-0.02),p=0.015** |
| **Crowding Index** | -0.01(-0.02,0.001) | 0.001(-0.001,0.003) | 0.01(-0.01,0.02) | 0.001(-0.01,0.01) |
| **Food basket** (Yes) | 0.09(-0.002,0.18) | **0.02(0.001,0.03),p=0.039** | **0.09(0.02,0.15),p=0.008** | 0.15(0.10,0.21), **p<0.001** |
| **Household food insecurity status** |  |  |  |  |
| Non-severly food insecure (Ref.) | --- | --- | --- | --- |
| Severly food insecure | 0.01(-0.07,0.10) | -0.01(-0.02,0.01) | 0.08(-0.05,0.21) | 0.06(-0.02,0.14) |
| † Variables adjusted for in the three models testing the impact of group status were variables found significantly different at baseline between IG and CG. These variables include school year, mother and father’s educational levels, crowding index, receiving assistance (food fasket), and household food insecurity status.  ¶ Weight for age z-scores were assessed only for children ≤ 10 years old (n = 68) [47]. | | | | |
