# Supplementary material for: Impact of a Pilot School-Based Nutrition Intervention on Dietary Knowledge, Attitudes, Behavior and Nutritional Status of Syrian Refugee Children in the Bekaa, Lebanon
Source: Nutrients. 2018 Jul 17;10(7):913. doi: 10.3390/nu10070913 (PMC6073287; doi:10.3390/nu10070913)
Supplement: Supplementary file 1 [file nutrients-10-00913-s001.zip › supplementary material/Table S4.docx]

**Table S4.** Within-group differences (baseline versus follow up) in mean change of nutrition knowledge, attitude, behavior scores and anthropometric measures of school-aged children (n=183).

|  | Intervention(n=102) | |  | Control(n=81) | |  |
| --- | --- | --- | --- | --- | --- | --- |
|  | **Baseline** | Follow up |  | **Baseline** | **Follow up** |  |
|  | **Mean ± SE** | | p-value^†^ | **Mean ± SE** | | p-value^†^ |
| Knowledge Attitude Behavior (KAB) scores^§^ |  |  |  |  |  |  |
| Knowledge scores | 9.98±0.18 | 12.23±0.14 | 0.004 | 9.76±0.25 | 10.64±0.21 | 0.002 |
| Attitude scores | 8.19 ±0.13 | 9.12±0.09 | 0.033 | 7.50±0.19 | 8.26± 0.14 | 0.001 |
| Behavior scores | 9.99±0.36 | 10.31±0.34 | 0.907 | 8.51±0.42 | 8.83±0.422 | 0.222 |
| Anthropometric measurements |  |  |  |  |  |  |
| BMI for Age Z-score (BAZ) | 0.12±0.10 | 0.22±0.10 | 0.773 | -0.04±0.11 | -0.14±0.11 | 0.265 |
| Height for age Z-score (HAZ) | -0.24±0.11 | 0.16±0.12 | 0.952 | -0.72±0.11 | -0.48±0.11 | 0.007 |
| Weight for age Z-score (WAZ)^¶^ | 0.36±0.16 | 0.70±0.17 | 0.706 | -0.30±0.18 | -0.21±0.19 | 0.047 |
| Waist to Height ratio (WHtR) | 0.48±0.006 | 0.47±0.006 | 0.041 | 0.47±0.005 | 0.46±0.006 | 0.011 |
| † Paired t-tests were conducted to compare KAB scores and anthropometrics between baseline and follow up within intervention and control groups. Statistical significance was determined at p-value <0.05.  § The total knowledge , behavior and attitude scores ranged : 0-15 points, 0-10 points and 0-22 points , respectively  ¶ Weight for age z-scores were assessed only for children ≤ 10 years old (n = 68) [51]. | | | | | | |
