# Supplementary material for: Impact of a Pilot School-Based Nutrition Intervention on Dietary Knowledge, Attitudes, Behavior and Nutritional Status of Syrian Refugee Children in the Bekaa, Lebanon
Source: Nutrients. 2018 Jul 17;10(7):913. doi: 10.3390/nu10070913 (PMC6073287; doi:10.3390/nu10070913)
Supplement: Supplementary file 1 [file nutrients-10-00913-s001.zip › supplementary material/Table S5.docx]

**Table S5.** Within-group differences (baseline versus follow up) in mean change of total energy (kcal/day) and macro- and micronutrient intakes (g/day) among school-aged children (n=178).

|  | Intervention (n=99) | |  | Intervention(n=79) | |  |
| --- | --- | --- | --- | --- | --- | --- |
|  | Baseline | **Follow up** |  | Baseline | **Follow up** |  |
|  | Mean ± SE | | p-value^†^ | Mean ± SE | | p-value^†^ |
| Energy intake (Kcal) | 1,199.82±46.09 | 1,287.17±62.99 | 0.168 | 1,325.41±47.69 | 1,217.24±45.14 | 0.099 |
| Macronutrients |  |  |  |  |  |  |
| Carbohydrates (g) | 153.27±6.69 | 160.18±7.86 | 0.072 | 170.39±7.42 | 162.76±6.98 | 0.189 |
| Sugar (g) | 32.15±2.60 | 29.88±2.75 | 0.692 | 29.06±2.36 | 31.15±2.38 | 0.625 |
| Dietary fiber (g) | 11.74±0.65 | 13.13±0.76 | 0.400 | 13.79±0.70 | 11.39±0.56 | 0.046 |
| Protein (g) | 28.88±1.28 | 34.39±1.81 | 0.087 | 29.08±1.32 | 28.44±1.44 | 0.921 |
| Total fat (g) | 53.62±2.78 | 57.78±3.59 | 0.504 | 59.89±2.90 | 51.40±2.54 | 0.011 |
| MUFA (g) | 24.62±1.62 | 26.90±2.05 | 0.542 | 29.02±1.72 | 23.80±1.40 | 0.006 |
| PUFA (g) | 13.18±0.89 | 12.72±0.82 | 0.932 | 14.79±0.89 | 13.06±0.82 | 0.050 |
| Saturated fat (g) | 8.78±0.51 | 10.35±0.78 | 0.190 | 10.48±0.71 | 8.77±0.57 | 0.030 |
| Trans fat (g) | 0.15±0.03 | 0.096±0.02 | 0.732 | 0.11±0.03 | 0.13±0.02 | 0.678 |
|  |  |  |  |  |  |  |
| Micronutrients |  |  |  |  |  |  |
| Vitamin C (mg) | 35.24±4.22 | 40.97±4.39 | 0.273 | 24.36±2.20 | 25.86±3.69 | 0.662 |
| Vitamin A (µg) | 125.43±45.21 | 215.35±22.96 | 0.535 | 88.55±12.73 | 88.35±10.45 | 0.077 |
| Vitamin D (µg) | 0.31±0.06 | 0.65±0.19 | 0.178 | 0.33±0.10 | 0.47±0.16 | 0.834 |
| Vitamin E (mg) | 7.50±0.55 | 7.71±0.51 | 0.969 | 8.16±0.45 | 6.62±0.40 | 0.006 |
| Vitamin K (µg) | 110.30±9.37 | 226.39±19.59 | 0.057 | 153.34±11.20 | 118.24±9.55 | 0.516 |
| Vitamin B1 (mg) | 1.02±0.05 | 1.10±0.05 | 0.044 | 1.08±0.06 | 1.06±0.06 | 0.667 |
| Vitamin B2 (mg) | 1.01±0.21 | 0.97±0.096 | 0.941 | 0.93±0.15 | 0.76±0.04 | 0.469 |
| Vitamin B3 (mg) | 7.61±0.54 | 8.81±0.71 | 0.482 | 7.07±0.45 | 7.11±0.54 | 0.594 |
| Vitamin B6 (mg) | 0.65±0.04 | 0.72±0.05 | 0.197 | 0.61±0.04 | 0.59±0.04 | 0.743 |
| Vitamin B12 (µg) | 0.64±0.23 | 0.65±0.11 | 0.617 | 0.39±0.07 | 0.63±0.11 | 0.346 |
| Folate (µg) | 180.41±13.89 | 200.25±13.68 | 0.474 | 152.34±12.79 | 135.43±10.97 | 0.717 |
| Iron (mg) | 8.83±0.58 | 9.88±0.64 | 0.660 | 11.27±0.56 | 8.88±0.57 | 0.003 |
| Zinc (mg) | 4.44±0.30 | 4.74±0.30 | 0.252 | 6.47 ±0.33 | 4.86±0.30 | 0.004 |
| Calcium (mg) | 293.84±18.27 | 433.29±33.59 | 0.051 | 349.15±19.95 | 323.51±26.85 | 0.842 |
| Phosphorus (mg) | 440.32±23.39 | 524.20±33.07 | 0.147 | 450.41±22.96 | 435.11±23.92 | 0.680 |
| Magnesium (mg) | 135.81±7.01 | 168.25±9.50 | 0.156 | 148.78±6.03 | 131.29±5.70 | 0.193 |
| Sodium (g) | 1.47±0.07 | 1.70±0.11 | 0.149 | 1.37±0.78 | 1.39±0.07 | 0.655 |
| Potassium (g) | 1.27±0.07 | 1.43±0.09 | 0.251 | 1.19±0.07 | 1.16±0.06 | 0.857 |
| † Paired t-tests were conducted to compare nutrient intakes between baseline and follow up within intervention and control groups. Statistical significance was determined at p-value <0.05. | | | | | | |
