# Supplementary material for: Impact of a Pilot School-Based Nutrition Intervention on Dietary Knowledge, Attitudes, Behavior and Nutritional Status of Syrian Refugee Children in the Bekaa, Lebanon
Source: Nutrients. 2018 Jul 17;10(7):913. doi: 10.3390/nu10070913 (PMC6073287; doi:10.3390/nu10070913)
Supplement: Supplementary file 1 [file nutrients-10-00913-s001.zip › supplementary material/Table S6.docx]

**Table S6.** Post-intervention nutritional status of school-aged children enrolled in intervention and control elementary schools in the Bekaa region, Lebanon (n=183).

|  | **Intervention** | | **Control** | |
| --- | --- | --- | --- | --- |
|  | **Baseline** | **Follow up** | **Baseline** | **Follow up** |
|  | **n (%)** | | **n (%)** | |
| BMI-for-age Z scores (BAZ) |  |  |  |  |
| Thin (BAZ ≤ -2) | 2 (1.8) | 1 (0.9) | 3 (3.4) | 4 (4.5) |
| Normal (-2 <BAZ ≤ +1) | 87 (77.7) | 87 (77.7) | 74 (84.1) | 76 (86.4) |
| Overweight (+1< BAZ ≤ +2) | 14 (12.5) | 15 (13.4) | 8 (9.1) | 5 (5.7) |
| Obese ( BAZ >+2) | 9 (8.0) | 9 (8.0) | 3 (3.4) | 3 (3.4) |
